# Multi-Trait Multi-Environment Genomic Prediction of Agronomic Traits in Advanced Breeding Lines of Winter Wheat

**DOI:** 10.3389/fpls.2021.709545

**Published:** 2021-08-18

**Authors:** Harsimardeep S. Gill, Jyotirmoy Halder, Jinfeng Zhang, Navreet K. Brar, Teerath S. Rai, Cody Hall, Amy Bernardo, Paul St Amand, Guihua Bai, Eric Olson, Shaukat Ali, Brent Turnipseed, Sunish K. Sehgal

**Affiliations:** ^1^Department of Agronomy, Horticulture & Plant Science, South Dakota State University, Brookings, SD, United States; ^2^Department of Plant Pathology, Kansas State University, Manhattan, KS, United States; ^3^United States Department of Agriculture - Agricultural Research Services, Hard Winter Wheat Genetic Research Unit, Manhattan, KS, United States; ^4^Department of Plant, Soil and Microbial Sciences, Michigan State University, East Lansing, MI, United States

**Keywords:** BMTME, GBS, genomic prediction, genomic selection, G × E, multi-trait multi-environment genomic prediction, wheat breeding

## Abstract

Genomic prediction is a promising approach for accelerating the genetic gain of complex traits in wheat breeding. However, increasing the prediction accuracy (PA) of genomic prediction (GP) models remains a challenge in the successful implementation of this approach. Multivariate models have shown promise when evaluated using diverse panels of unrelated accessions; however, limited information is available on their performance in advanced breeding trials. Here, we used multivariate GP models to predict multiple agronomic traits using 314 advanced and elite breeding lines of winter wheat evaluated in 10 site-year environments. We evaluated a multi-trait (MT) model with two cross-validation schemes representing different breeding scenarios (CV1, prediction of completely unphenotyped lines; and CV2, prediction of partially phenotyped lines for correlated traits). Moreover, extensive data from multi-environment trials (METs) were used to cross-validate a Bayesian multi-trait multi-environment (MTME) model that integrates the analysis of multiple-traits, such as G × E interaction. The MT-CV2 model outperformed all the other models for predicting grain yield with significant improvement in PA over the single-trait (ST-CV1) model. The MTME model performed better for all traits, with average improvement over the ST-CV1 reaching up to 19, 71, 17, 48, and 51% for grain yield, grain protein content, test weight, plant height, and days to heading, respectively. Overall, the empirical analyses elucidate the potential of both the MT-CV2 and MTME models when advanced breeding lines are used as a training population to predict related preliminary breeding lines. Further, we evaluated the practical application of the MTME model in the breeding program to reduce phenotyping cost using a sparse testing design. This showed that complementing METs with GP can substantially enhance resource efficiency. Our results demonstrate that multivariate GS models have a great potential in implementing GS in breeding programs.

## Introduction

Global wheat production needs to be increased by 60% to meet the demand of a projected population of 9 billion by 2050 (Tester and Langridge, 2010; Fischer et al., 2014). In the past few decades, wheat breeding successfully achieved a significant increase in grain yield owing to significantly improved genetic resources, implementation of modern agronomic practices, accurate experimental designs, and other improved technology packages (Tadesse et al., 2019), which translates into an annual increase of 1% in terms of genetic gain in grain yield. However, this increase is still far from the expected yearly growth of 1.7% to meet the future wheat demand (Oury et al., 2012; Tadesse et al., 2019). Thus, new and innovative breeding technologies are essential to achieve a 2-fold increase in annual yield to avoid potential food crises in the coming decades.

Traditional wheat breeding involves creating novel genetic variation with different methods, followed by extensive selection and advancement of generations. The selection of progeny with desirable agronomic and end-use quality traits is a resource-intensive process and could take up to 10–15 years to develop a new cultivar (Haile et al., 2020). Further, in traits with complex genetic architecture such as grain yield, genotype-by-environment interactions play a paramount role and impose additional challenges in selection. In recent years, the deployment of molecular markers for marker-assisted selection (MAS) has been used to increase the selection accuracy and accelerate genetic gain (Randhawa et al., 2013). Although MAS has shown a good potential in wheat breeding for the deployment of qualitative trait loci (QTLs) with large effects, its application has been limited to improve complex traits governed by many QTLs with small effects (Heffner et al., 2009).

Genomic selection (GS) is a recent approach that utilizes genome-wide marker data to select individuals superior for complex traits in the early breeding cycle to increase the genetic gain per unit of time (Meuwissen et al., 2001; Heffner et al., 2009). Unlike MAS, GS does not require prior identification of QTLs for traits of interest; instead, it employs all available markers across the genome to predict breeding values of individuals (Bassi et al., 2015). Briefly, GS requires a training population (TP), which is genotyped with genome-wide markers and for a given trait(s) of interest. GS involves the calibration of a prediction model using the TP to estimate marker effects and evaluate the predictive ability of the model through cross-validation. Finally, the developed model is used to calculate genome-estimated breeding values (GEBVs) and rank the lines from a breeding or testing population (BP) that consists of lines with only genotypic information. Thus, the early selection or culling of individuals based on GEBVs permits greater genetic gain per breeding cycle, facilitating an increase in the efficacy of breeding programs and resulting in reduced varietal development costs. Several studies have reported the successful implementation of GS in different crops resulting in an accelerated rate of genetic gain compared with traditional breeding (Bassi et al., 2015; Battenfield et al., 2016; Bhat et al., 2016). Moreover, GS has shown to be particularly useful in traits where phenotyping is cumbersome, such as quality traits and complex resistance to diseases (Battenfield et al., 2016; Dong et al., 2018).

The widespread availability of genome-wide markers attributed to low-cost genotyping technologies has facilitated the adaptability of GS in wheat breeding programs (Poland et al., 2012b; Bhat et al., 2016). Thus, in recent years, there has been a growing interest to complement phenotyping selection and genomic selection in wheat breeding. GS has been evaluated for many complex traits in wheat, including but not limited to grain yield and yield-related traits (Rutkoski et al., 2016; Ward et al., 2019; Guo et al., 2020; Haile et al., 2020; Juliana et al., 2020), wheat resistance to rusts (Rutkoski et al., 2014; Juliana et al., 2017), Fusarium head blight (Rutkoski et al., 2012; Arruda et al., 2015; Dong et al., 2018), and end-use quality traits (Battenfield et al., 2016; Lado et al., 2018; Ibba et al., 2020). Despite the successful evaluations of GS in wheat breeding programs, there is a continuous scope to improve the prediction accuracy/ability of GS models for quantitative traits to achieve higher genetic gains that will lead to the routine implementation of GS in various wheat breeding schemes.

The predictive ability of the genomic selection model refers to the correlation between estimated genome-estimated breeding values and the actual phenotypic values of individuals in a validation set and is generally calculated through a cross-validation approach. Along with TP size, extent of linkage disequilibrium (LD), and heritability of traits, predictive ability also depends on the choice and optimization of statistical models (de los Campos et al., 2013; Rutkoski et al., 2016; Guo et al., 2020). In most studies, penalized genomic prediction models, such as ridge-regression best linear unbiased prediction (rrBLUP) and genomic best linear unbiased prediction (GBLUP), have been standard GS approaches (VanRaden et al., 2009; Endelman, 2011). In addition, several Bayesian methods with different prior distributions and relying on Markov-Chain Monte Carlo (MCMC) for estimation of parameters have proven useful for genomic prediction (Habier et al., 2011; Wang et al., 2018). However, most of these models implement a univariate linear mixed model and are helpful in predicting only one variable at a time.

In recent years, multi-trait genomic prediction models have been suggested to improve the PA for a primary trait when secondary traits correlated to the primary trait are available (Jia and Jannink, 2012). The use of genetically correlated traits is of particular importance when the primary trait is difficult or expensive to phenotype and has low heritability. Several empirical studies have successfully evaluated multi-trait (MT) approaches for different agronomic traits in wheat breeding (Rutkoski et al., 2012; Hayes et al., 2017; Lado et al., 2018). An improvement of 70% in PA for grain yield was observed by including canopy temperature (CT) and normalized difference vegetation index as secondary traits using the MT approach (Rutkoski et al., 2016; Sun et al., 2017). Similarly, Hayes et al. (2017) and Lado et al. (2018) observed an increase in PA using multivariate approaches (MT) over single trait (ST) models in end-use quality traits.

For complex traits, genotype-by-environment (G × E) interactions necessitate the evaluation of breeding lines for multiple traits over multiple environments. Thus, the extension of MT approaches to account for a G × E interaction could improve the model for genomic prediction accuracy in breeding programs. Montesinos-López et al. (2016) proposed a Bayesian multi-trait and multi-environment (BMTME) model that integrates the analysis of multi-traits recorded over multi-environments in a unified approach. Recently, an improved BMTME model has been introduced that estimates the variance-covariance structure among traits, genotypes, and environments to predict multiple traits evaluated in various environments (Montesinos-López et al., 2019). Some studies using simulated and empirical data found that the BMTME model outperforms ST models in agronomic and end-use quality traits in wheat (Montesinos-López et al., 2016; Guo et al., 2020; Ibba et al., 2020). The better performance of multivariate GS approaches stimulates us to evaluate these models in an actual breeding pipeline, where several traits are evaluated over diverse environments.

Although different GS approaches have been tested for predicting complex traits in wheat breeding programs, only few studies have reported the application of GS in actual yield trials where lines are evaluated over several environments (Belamkar et al., 2018). GS has a great potential in the early selection or culling in preliminary trials using information from advanced trials and accelerates genetic improvement. Furthermore, GS can complement phenotypic selection in practical scenarios such as loss of complete/partial trials due to weather extremes. In this study, we focused on the use of advanced breeding lines evaluated over multiple environments as training sets to predict untested genotypes using univariate and multivariate GS approaches. The specific objectives of this study were to (1) estimate the PA of various agronomic traits in advanced breeding lines using univariate and multivariate GP models and different cross-validation schemes, (2) assess the reliability of multivariate GP models in predicting complex traits over different years and locations, and (3) investigate the application of multi-trait multi-environment GP models in sparse testing of breeding lines.

## Materials and Methods

### Plant Materials

The experiment was conducted for two growing seasons (2018–19 and 2019–20) using a total of 314 winter wheat genotypes. The genotypes included breeding lines from 2018 to 2019 and 2019 to 2020 wheat advanced yield trials (AYTs) and elite yield trials (EYTs) from the South Dakota State University (SDSU) winter wheat breeding program and well-adapted check cultivars. Most of the genotypes were either F_4:7_ or F_4:8_ filial generation. Of the 314 genotypes, 157 were evaluated in the growing season of 2019 and another 157 in that of 2020. Forty-four genotypes were shared between the two sets of wheat materials, leaving 270 unique genotypes in the study. We removed seven genotypes from genomic prediction analyses because of low-quality genotypic data. Thus, 151 and 156 genotypes were used for further analyses in the 2018–19 and 2019–20 growing seasons, respectively.

### Experimental Design and Trait Measurement

The experimental plots were planted under a no-till system at five locations in South Dakota (Supplementary Table 1) in both seasons. The experimental unit at each of the five locations consisted of 1.5-m wide and 4-m long plots with seven rows spaced 20 cm apart. The seeding rate for plots was 300 seeds m^−2^ at all the locations. Recommended agronomic practices were followed for proper growth and yield.

Five agronomic traits were measured in this study, namely, grain yield (YLD) (bushels acre^−1^), grain protein content (PROT) (%), test weight (TW) (kg hL^−1^), plant height (HT) (cm), and days to heading (HDs) (Julian days). YLD was determined after harvesting the plots upon maturity using a plot combine (Zurn, Westernhausen Germany). PROT, TW, and moisture content were measured using Infratec^TM^ 1241 Grain Analyzer (FOSS North America, Eden Prairie, MN, United States). YLD from plot and PROT were adjusted to 13% moisture content equivalence. HT was recorded as the distance from the soil surface to the tip of the fully emerged spike, excluding any awns if present. HDs were recorded as the Julian days required for 50% of heads to emerge from the boot in each plot.

### Phenotypic Data Analysis

The phenotypic data for all the five agronomic traits were analyzed using best linear unbiased estimates (BLUEs) for individual environments. The model used for estimation of the genotypic BLUEs for individual environments was as follows:

(1) $$\begin{array}{r} y_{ij}=\mu +R_{i}+G_{j}+e_{ij} \end{array}$$

where y_*ij*_ is the trait of interest, μ is the overall mean, R_*i*_ is the effect of the i^th^ replicate, G_*j*_ is the effect of the j^th^ genotype, and e_*ij*_ is the residual error effect associated with the i^th^ replication and j^th^ genotype. The replicates correspond to the complete blocks.

For the across environment estimation of best linear unbiased estimates (BLUEs) and best linear unbiased predictions (BLUPs), the statistical model was modified, as shown below:

(2) $$\begin{array}{r} y_{ijk}=\mu +E_{i}+R_{j\left(i\right)}+G_{k}+GE_{ik}+e_{ijk} \end{array}$$

where y_*ijk*_ is the trait of interest, μ is the overall mean, E_*i*_ is the effect of the i^th^ environment, R _*j*(*i*)_ is the effect of the j^th^ replicate nested within the i^th^ environment, G_*k*_ is the effect of the k^th^ genotype, GE_*ik*_ is the effect of the genotype × environment (G × E) interaction, and e_*ijk*_ is the residual error effect associated with the i^th^ replication and j^th^ genotype. The environment corresponds to the individual locations and replicates correspond to the complete blocks. The genotype was assumed as a fixed effect, whereas the environment and block nested within the environment were assumed as random effects.

The broad-sense heritability (*H*^2^) of a trait of interest in an independent environment was assessed as follows:

(3) $$\begin{array}{r} H^{2}=\;\frac{\sigma_{g}^{2}}{\sigma_{g}^{2}+\;\sigma_{e}^{2}/nRep} \end{array}$$

where $\sigma_{g}^{2}$and $\sigma_{e}^{2}$ are the genotype and error variance components, respectively. The BLUEs and variance components were estimated using META-R (Alvarado et al., 2020), which employs the LME4 R-package (Bates et al., 2015) for linear mixed model analysis. The Pearson correlations among traits and environments were estimated based on the BLUEs and BLUPs using the “psych” package in the R environment (R Core Team, 2018). The genetic correlations between the five traits were estimated for individual years using the “BMTME” R package (Montesinos-López et al., 2019).

### SNP Genotyping

Fresh leaf tissues were collected from each line for DNA isolation using the hexadecyltrimethylammonium bromide (CTAB) method (Doyle and Doyle, 1987). Genotyping-by-sequencing (GBS) was performed following double digestion with HF-*PstI* and *MspI* restriction enzymes for library preparation (Poland et al., 2012a). GBS libraries were sequenced using an IonProton sequencer (Thermo Fisher Scientific, Waltham, MA, United States) at the USDA Central Small Grain Genotyping Lab, Manhattan, KS, United States. TASSEL v5.0 was used to call single-nucleotide polymorphisms (SNPs) using the GBS v2.0 discovery pipeline (Bradbury et al., 2007). The reads were aligned to the Chinese Spring wheat genome reference RefSeq v1.1 (IWGSC, 2018) using the default settings of Burrows–Wheeler Aligner v0.6.1. For quality control, SNPs with more than 20% missing data points and minor allele frequency (MAF) of <0.05 were removed. Finally, we obtained 10,290 high-quality SNPs after removing the SNPs that were unmapped on any wheat chromosome. The missing data points in the selected SNP set were imputed using BEAGLE v4.1 (Browning and Browning, 2007). The additive relationship matrix for GP models was estimated using the *A.mat* function in the “rrBLUP” package in R (Endelman, 2011). The Kinship (K)-based marker matrix was estimated using the Centered IBS (identity by state) method (Endelman and Jannink, 2012) implemented through Genomic Association and Prediction Integrated Tool (GAPIT) (Tang et al., 2016).

### Genomic Prediction Models and Cross-Validation

We evaluated one univariate and two multivariate GP models for predicting five agronomic traits. Different cross-validation schemes that mimic actual scenarios in a breeding program were used to estimate the PA of these traits and compare the performance of different models.

#### Single-Trait Model

Ridge regression best linear unbiased prediction (Endelman, 2011) is the commonly used GS model in plant breeding. Similar to the genomic best linear unbiased prediction (GBLUP) model, rrBLUP assumes the normal distribution of marker effects with equal variance. We used rrBLUP as a baseline GS model for all the traits to evaluate the performance of multivariate models. The within-environment trait BLUEs were calculated and then used as input to perform rrBLUP within each environment. A linear mixed model was implemented using the following model:

(4) $$\begin{array}{r} y=1\mu +Zu+\varepsilon \end{array}$$

where *y* is the vector (*n* × *1*) of adjusted means (BLUEs) from *n* genotypes for a given trait; μ is the overall mean; *Z* is the design matrix *(n* × *p)* with known values of *p* markers for *n* genotypes; *u* is a genotypic predictor with *u* ~*N(*0, G_nxn_$\sigma_{g}^{2}$), where *G* is positive semi-definite matrix, obtained from markers using “*A.mat*,” which is an additive relation matrix function and $\sigma_{g}^{2}$ is the additive genetic variance; ε is the residual error with *e* ~*N(*0, $\sigma_{e}^{2}$).

#### Multi-Trait Model

A Bayesian Multivariate Gaussian model with an unstructured variance-covariance matrix was used for the multi-trait model (MT) (Lado et al., 2018). The MT model can be described as

(5) $$\begin{array}{r} y=1\mu +Zu+\varepsilon \end{array}$$

where *y* is the vector with a length of *n* × *t* (*n* genotypes and *t* traits); μ is the means vector; *Z* represents the incidence matrix of order [(*n* × *t*)*p*]; *u*_[(n × *t*)*p*]_ is a genotypic predictor for all individuals and traits with u ~*N*(0, ∑ ⊗ G). The matrix *G* represents the positive semi-definite matrix obtained from markers. The residuals of the MT model are represented by the vector ε, with ε ~*N*(0, R ⊗ I). The matrices ∑ and *R* are the variance-covariance matrices for depicting the genetic and residual effects, respectively, for each individual in all traits, estimated with the Gibbs sampler with 5,000 burn-in and 25,000 iterations in R package “MTM” (de los Campos and Grüneberg, 2016). The ∑ was estimated as an unstructured matrix and R as a diagonal matrix following Lado et al. (2018).

#### Bayesian Multi-Trait Multi-Environment Model

The Bayesian multi-trait multi-environment model for genomic predictions (Montesinos-López et al., 2016, 2019) can be briefly described as

(6) $$\begin{array}{r} y=X\beta +Z_{1}b_{1}+Z_{2}b_{2}+\varepsilon \end{array}$$

where *y* is the response matrix of order *j* × *t* (where *t* is the number of traits and *j*= *n* × *l*, where *n* denotes the number of genotypes and *l* denotes number of environments); *X* is the design matrix for environmental effects of order *n* × *l*, whereas β is the matrix of beta coefficients of order *l* × *t*. *Z*_1_ is the incidence matrix of genotypes of order *j* × *n*, and *b*_1_ is the matrix of genotypic random effects of order *n* × *t*. *Z*_2_ is the incidence matrix of genotype × environment interaction of order *j* × *ln* and *b*_2_ is the random effect of genotype × environment × traits of order *ln* × *t*. We assume that *b*_1_ is distributed under a matrix variate normal distribution as *b*_1_ ~*MN*(0, *G*, ∑_*t*_), where *G* is of order *n* × *n*, obtained from SNP markers using “*A.mat*,” which is an additive relation matrix function in rrBLUP, and ∑_*t*_ is the unstructured variance-covariance matrix of traits of order *t* × *t*. The *b*_2_ is assumed to be distributed under a matrix variate normal distribution as *b*_2_ ~*MN*(0, ∑_*E*_⊗G, ∑_*t*_), where ⊗ denotes a Kronecker product and ∑_*E*_ is the unstructured variance-covariance matrix of *l* × *l*. The matrix ε is the matrix of residuals of order *j* × *t* distributed as ε ~*MN*(0, *l*_*j*_, *R*_*e*_). A detailed account of this model and prior distributions can be found in Montesinos-López et al. (2019). Model simulations were carried out using the R package “BMTME” (Montesinos-López et al., 2019) with 5,000 burn-in and 25,000 iterations.

#### Assessment of Prediction Ability

Predictive ability was estimated as Pearson correlation coefficient between genome-estimated breeding values and observed phenotypes for the testing set of breeding lines. The PA for the rrBLUP model was estimated using cross-validation scheme 1 (CV1), where the population was equally divided into five subpopulations, with four subpopulations (80%) as the training population (phenotyped and genotyped) to train the model and one subpopulation (20%) as the testing population (genotyped only) for prediction. The single-trait model with cross-validation scheme 1 (designated as ST-CV1 hereafter) was implemented in the “rrBLUP” R package (Endelman, 2011) for one trait at a time. The cross-validation process was repeated 1,000 times, and each iteration included different lines in the training and testing sets.

The prediction accuracy (PA) of the MT model was estimated using two cross-validation schemes, as described in Lado et al. (2018) (Supplementary Figure 1). Similar to the ST-CV1 scheme, the first cross-validation scheme (MT-CV1) used a random set of lines (80%) as a training set and the remaining lines (20%) as a testing set. The model was trained using genotypic and phenotypic data of these lines in the training set, and only genotypic data were used to predict the performance of the testing set lines based on the model built from the training set. This process of splitting the data into training and testing sets was repeated 50 times. Hence, a different set of lines were selected into the training and testing datasets for each iteration. The CV1 scheme mocks the breeding situation where a set of lines that are evaluated for given traits could be used to predict an unphenotyped set of lines that only have genotypic information. In the second cross-validation scheme (MT-CV2), the lines were randomly split into a training set (80%) and a testing set (20%). To train the model, MT-CV2 used genotypic data and phenotypic data of secondary traits from both the training and testing sets, but the phenotypic data of the target trait (primary trait) only from the training set. The BMTME model used a cross-validation scheme similar to MT-CV1 to estimate the PA of the model by randomly splitting the lines into an 80% training set and a 20% testing set. Since the BMTME model employs a Gibbs sampler with multiple iterations and is computationally expensive, the cross-validation scheme was repeated only 25 times.

### Application of MTME Genomic Prediction in the Breeding Program

As the multi-trait multi-environment model showed a potential in predicting different agronomic traits the using cross-validation approach, we evaluated the possible application of this method in the breeding program to reduce phenotyping efforts and per plot costs. As discussed earlier, we evaluated ~40 elite lines and ~110 advanced lines each year in multiple environments. Per plot costs and phenotyping efforts could be reduced if we can successfully determine the genomic estimation of breeding values (GEBVs) of the advanced lines in fewer locations rather than testing these lines in all available locations. The MTME model can estimate the environmental effect based on elite lines evaluated in all locations and the genotypic effect of advanced lines from fewer locations. To test this, we used the MTME model in an allocation design where we used the phenotypic data of elite lines from five testing environments; however, we used phenotypic records of advanced lines from three environments only. We predicted five traits in the remaining two environments in both the growing seasons. The model was fitted using the R package “BMTME” (Montesinos-López et al., 2016, 2019) with 5,000 burn-in and 15,000 iterations. The observed phenotypic records from the remaining two environments were used to assess the predictive accuracy of the design.

## Results

### Descriptive Statistics

The phenotypic BLUEs for grain yield, grain protein content, test weight, plant height, and days to heading varied significantly among the different environments (Table 1). HYS produced the highest mean grain yield in both years, where BRK and WIN produced the lowest grain yield in 2018–19 and 2019–20, respectively. Broad-sense heritability (*H*^2^) was estimated for all the five agronomic traits in each environment (Table 1). Differences in heritability estimates (0.63–0.96) describe the different genetic architecture of traits and contrasting environmental effects. Among the five traits evaluated in the study, TW, HT, and HDs had moderate to high heritability values in most of the environments and over both years. Relatively, YLD (0.64–0.84) and PROT (0.63–0.96) had comparatively lower heritability than other traits. Among the five environments, the heritability for all the traits was high in both the experimental years in DL. For YLD heritability, HYS (2019–20) had the highest (0.84), whereas BRK (2019–20) had the lowest (Table 1).

**Table 1:** Trait descriptive statistics and broad-sense heritability estimate for individual site-year environments of lines grown over five locations (Env) in 2018–19 and 2019–20 growing seasons.

**Year**	**Env^a^**	**Yield** **(bu ac** ^****−1****^ **)**			**Protein content** **(%)**			**Test weight** **(kg hL** ^****−1****^ **)**			**Plant height** **(cm)**			**Days to heading** **(julian days)**		
		**GM^b^**	**CV**	**H^**2**^**	**GM**	**CV**	**H^**2**^**	**GM**	**CV**	**H^**2**^**	**GM**	**CV**	**H^**2**^**	**GM**	**CV**	**H^**2**^**
2018-19	BRK	64.69	8.96	0.80	12.16	5.45	0.69	72.51	1.42	0.91	94.78	4.25	0.89	163.22	0.48	0.92
	DL	77.44	6.48	0.77	14.25	1.55	0.94	77.09	1.35	0.78	86.06	3.99	0.89	164.65	0.76	0.92
	HYS	81.98	6.46	0.73	12.04	3.66	0.72	76.55	1.06	0.92	99.12	3.18	0.90	163.84	0.70	0.74
	OND	71.21	7.27	0.76	13.25	3.30	0.85	78.21	1.27	0.82	89.91	3.62	0.91	168.73	0.65	0.87
	WIN	81.27	5.89	0.79	13.17	4.27	0.63	79.12	1.00	0.88	93.63	2.90	0.95	164.19	0.80	0.89
2019-20	BRK	84.26	6.25	0.64	12.49	3.65	0.80	77.39	0.90	0.89	86.67	4.42	0.75	156.18	0.63	0.89
	DL	93.31	4.14	0.78	13.55	1.40	0.96	79.10	0.64	0.95	85.80	3.45	0.83	155.74	0.40	0.94
	HYS	96.64	4.66	0.84	13.87	2.02	0.90	77.26	1.30	0.91	102.8	3.95	0.82	159.36	0.51	0.85
	OND	92.21	4.40	0.81	11.99	4.97	0.59	78.65	1.08	0.87	92.53	3.59	0.85	157.09	0.63	0.87
	WIN	84.16	4.73	0.80	13.24	2.99	0.84	78.24	0.90	0.89	92.70	3.47	0.85	158.75	0.63	0.91

*BRK, Brookings; DL, Dakota Lakes; HYS, Hayes; OND, Onida; and WIN, Winner*.

a *Env, refers to different trial location. BRK, Brookings; DL, Dakota Lakes; HYS, Hayes; OND, Onida; and WIN, Winner*.

b *GM, general mean for respective trait; CV, coefficient of variation; H^2^, broad sense heritability*.

**Figure 1:**
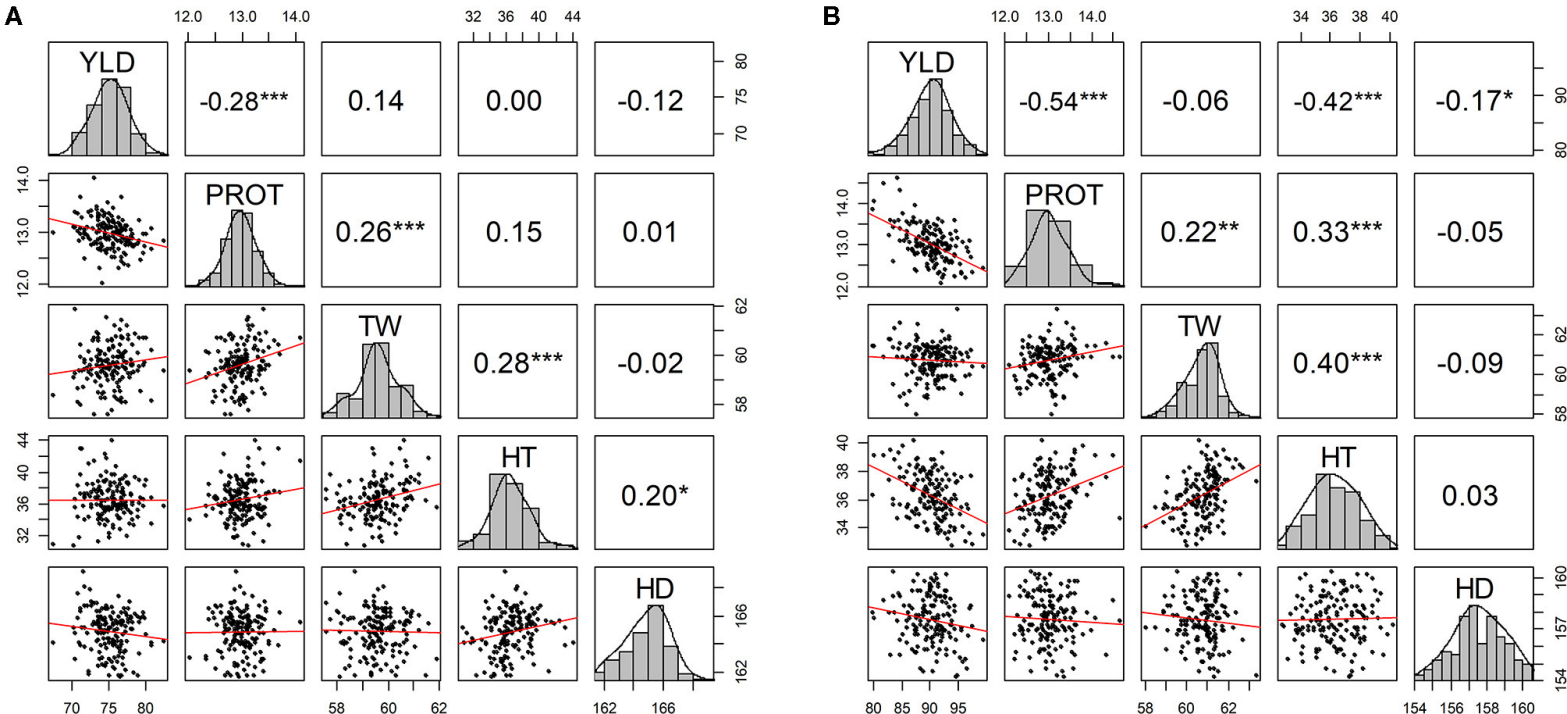
Scatter plot matrix with phenotypic distributions and Pearson correlations between agronomic traits using best linear unbiased predictions (BLUPs) by combining five experimental sites (BRK, DL, HYS, OND, and WIN) **(A)** from the growing season of 2018–19 and **(B)** from the growing season of 2019–20. YLD, grain yield; PROT, grain protein content; TW, test weight; HT, plant height; and HD, days to heading. *P ≤ 0.05, **P ≤ 0.01, ***P ≤ 0.001, ****P ≤ 0.0001.

Pearson correlations among agronomic traits were calculated using BLUEs by combining phenotypic data from all environments in each of the two growing seasons (Figure 1). As expected, significant negative correlation values (−0.28 and −0.54) were observed between YLD and PROT in both years. YLD was also negatively correlated with HDs (in both years) and HT (2019–20) (Figure 1). Similarly, TW was positively correlated with PROT and HT in both growing seasons. Overall, higher correlation values were observed between the agronomic traits in the 2019–20 growing season than in 2018–19 (Supplementary Figures 2, 3). Furthermore, genetic correlations among the five traits are estimated by fitting the BMTME model for individual growing seasons and are presented in Supplementary Tables 2, 3. Similar to the phenotypic correlation estimates, we observed a higher genetic correlation in 2019–20 as compared to 2018–19.

**Figure 2:**
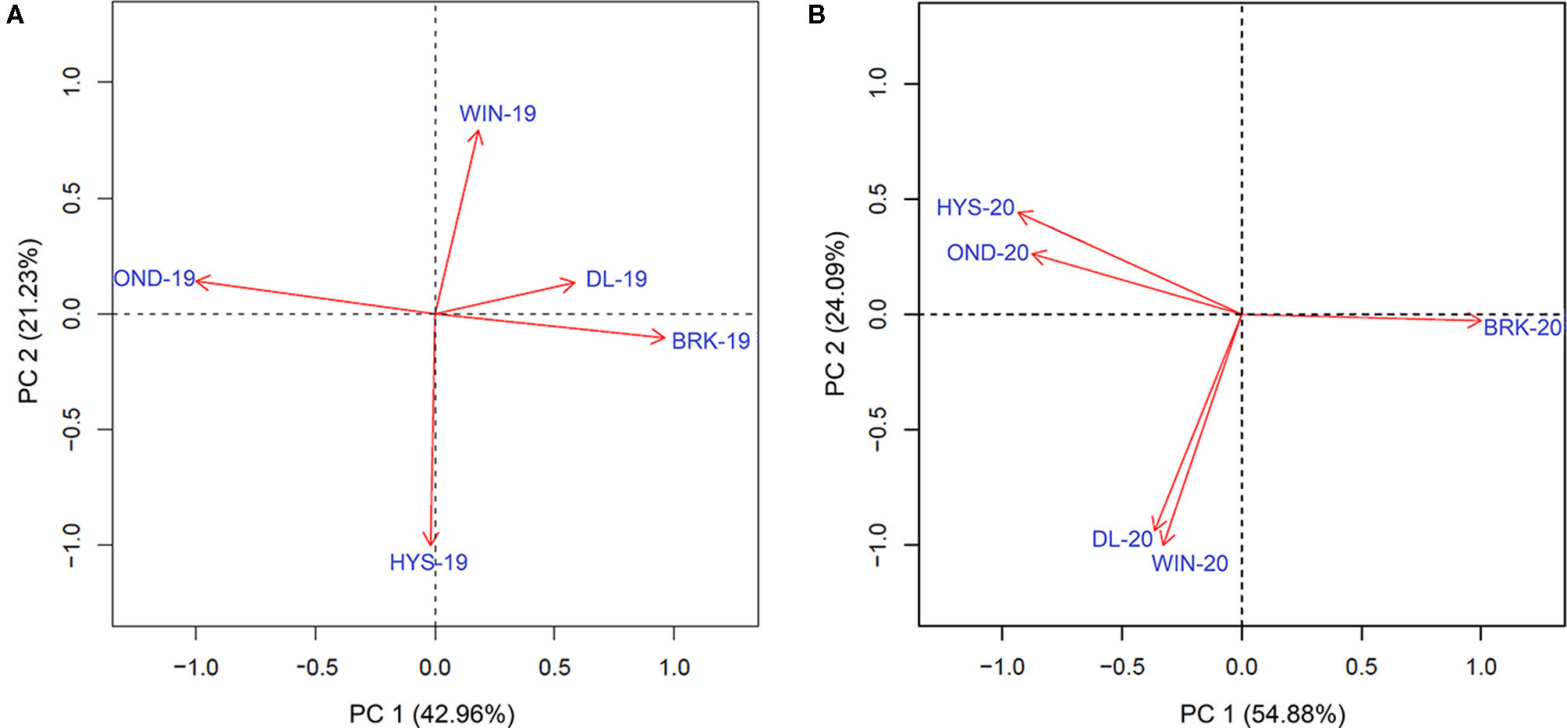
Principal component analysis to determine the association of the observed grain yield among five different experimental sites in the **(A)** 2018–19 growing season and the **(B)** 2019–20 growing season. BRK, Brookings; DL, Dakota Lakes; HYS, Hayes; OND, Onida; and WIN, Winner.

We further estimated the Pearson correlations among the five environments in 2018–19 and 2019–20 using the data of all the five agronomic traits (Supplementary Figure 4). Significantly higher correlation values were observed for YLD among the five environments in 2019–20 than those in 2018–19. A similar trend was observed for PROT, TW, and HDs; however, correlations were comparable for HT between the two growing seasons (Supplementary Figure 4). Moreover, the principal component analysis (PCA) on YLD validated strong correlations among the testing locations, in particular between HYS and OND and between DL and WIN, in the 2019–20 growing season (Figure 2). However, only a weak correlation was observed between DL and BRK in the 2018–19 growing season. The varying degrees of correlation among the locations in different growing seasons provide an opportunity to compare the performance of the MTME model in different growing environments.

### Genetic Relationship Among Lines

The kinship-based marker relationship matrix was derived using 10,290 SNPs from 151 lines evaluated in the 2018–19 growing season and 156 lines evaluated in the 2019–20 growing season (Supplementary Figure 5). The positive values of the relationship matrix signify an increased likelihood of the allele from one line being detected in other lines. The heatmaps of both the relationship matrices elucidate several small groups of closely related individuals over both the growing seasons. Most of the lines seem genetically related to several others. However, the heatmaps did not reveal any large genetically structured sub-populations in either set of 151 or 156 lines, respectively. Thus, the absence of a strong structure suggests no advantage of performing stratified sampling for cross-validation schemes to estimate PA. Furthermore, the density of heatmaps revealed a closer relationship among the 156 lines evaluated in 2019–20 (Supplementary Figure 5A) than among the 151 lines evaluated in 2018–19 (Supplementary Figure 5B).

### Genomic Prediction Using 2018–19 and 2019–20 Datasets

**Figure 3:**
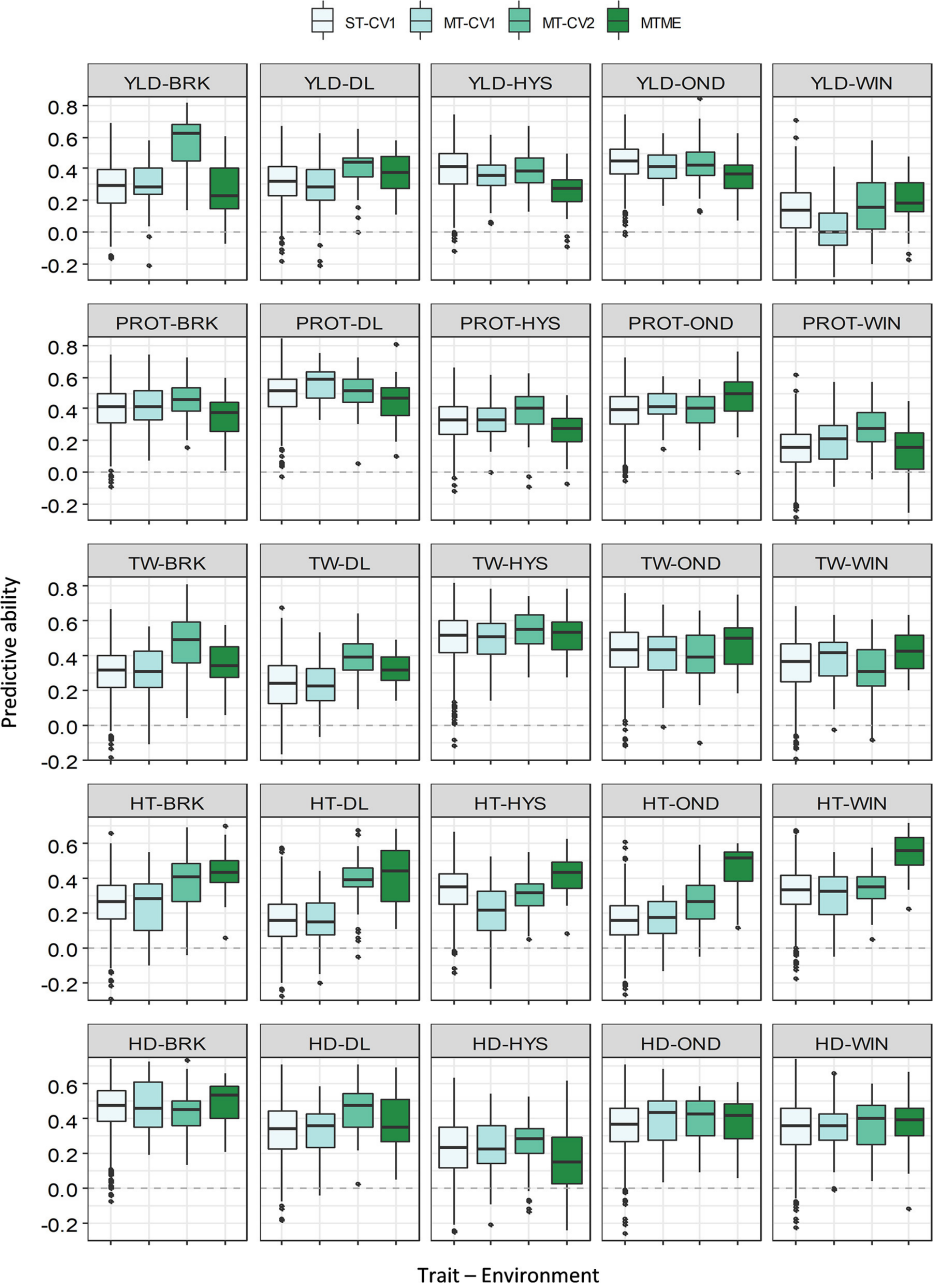
Prediction accuracy (PA) for five agronomic traits evaluated in five environments in the growing season of 2018–19. Boxplots compare the PA using a single-trait prediction model with one cross-validation scheme (ST-CV1), a multi-trait prediction model with two cross-validation schemes (MT-CV1 and MT-CV2), and a Bayesian multi-trait multi-environment prediction model (MTME). Traits include YLD, grain yield; PROT, grain protein content; TW, test weight; HT, plant height; and HD, days to heading.

We compared the predicted performance of five traits among four different approaches using two data sets (2018–19 and 2019–20). The PA of various models for the five traits is presented in Supplementary Tables 4, 5. ST-CV1 was used as a baseline model to compare the performance of different multivariate models. In 2018–19, the mean PA using ST-CV1 was 0.31, 0.35, 0.36, 0.35, and 0.36 for YLD, PROT, TW, HT, and HDs (Figure 3). Slightly better performance was observed in 2019–20 where ST-CV1 yielded an average PA of 0.36, 0.35, 0.54, 0.33, and 0.35 for these traits, respectively. The multi-trait model was tested using two prediction scenarios, MT-CV1 and MT-CV2. The MT-CV1 model did not show improvement in the PA over ST-CV1 for any of the five traits in either growing season (Supplementary Tables 4, 5).

The multi-trait model MT-CV2, which includes phenotypic data for secondary agronomic traits from individuals to be predicted, showed an overall higher prediction accuracy for YLD in both growing seasons. In 2018–19, the PA for YLD using the MT-CV2 model ranged from 0.15 to 0.56, outperforming the single-trait (ST-CV1) model by an average of 26% (Supplementary Tables 4, 5). Similarly, the mean PA for YLD in 2019–20 using MT-CV2 was 0.59, showing 63% improvement over the ST-CV1 model. The best PA for YLD in 2019–20 was observed in HYS (0.71), followed by WIN (0.67) and DL (0.57). The improvement in PA over ST-CV1 reached up to 148% in WIN and 80% in BRK in 2019–20.

**Figure 4:**
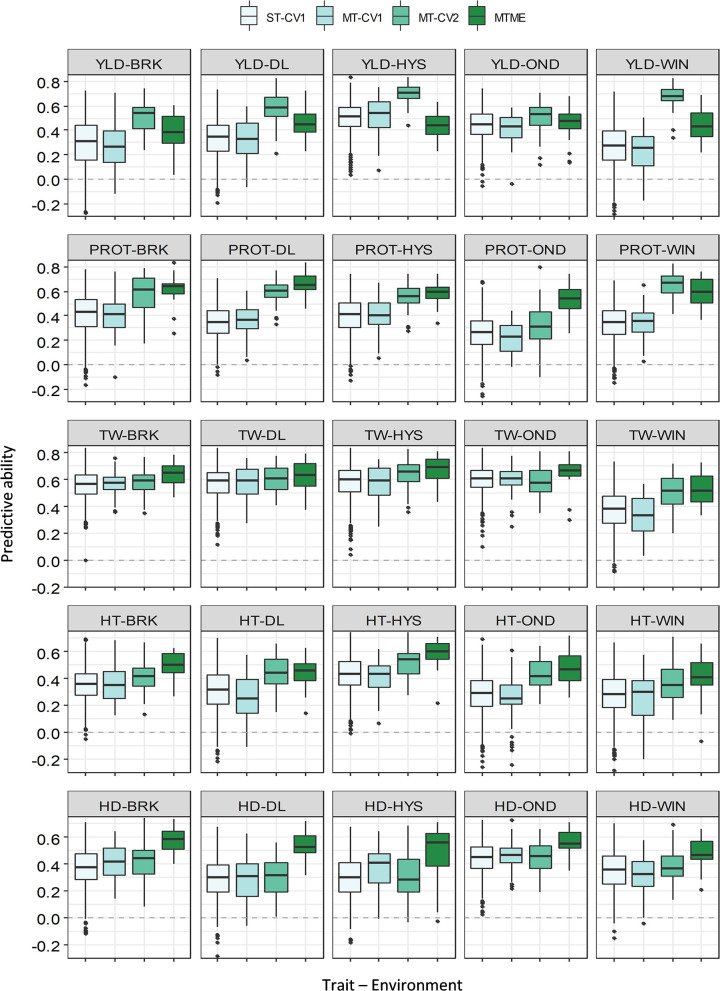
Prediction accuracy (PA) for five agronomic traits evaluated in five environments in the growing season of 2019–20. Boxplots compare the PA using a single-trait prediction model with one cross-validation scheme (ST-CV1), a multi-trait prediction model with two cross-validation schemes (MT-CV1 and MT-CV2), and a Bayesian multi-trait multi-environment prediction model (MTME). Traits include YLD, grain yield; PROT, grain protein content; TW, test weight; HT, plant height; and HD, days to heading.

Likewise, we observed a marginal to moderate improvement in PA for other agronomic traits using MT-CV2 model in both of the growing seasons (Figures 3, 4 and Supplementary Tables 4, 5). In 2018–19, the mean PA using MT-CV2 was 0.4, 0.42, 0.34, and 0.38 for PROT, TW, HT, and HDs, exhibiting an improvement of 14, 19, 36, and 8%, respectively. In comparison, the PA using MT-CV2 was higher in 2019–20, with an average PA of 0.54, 0.59, 0.43, and 0.38 for PROT, TW, HT, and HDs with an improvement of 54, 9, 30, and 8%, respectively. Overall, the better performance of the MT-CV2 model can be attributed to the higher genetic correlation among the traits evaluated in 2019–20 over the 2018–19 season (Supplementary Tables 2, 3).

The multi-trait multi-environment MTME model generalizes the multi-trait model to consider the correlation among the environments on top of the genetic correlation between the traits. In 2018–19, the MTME model did not show a significantly different PA over the ST-CV1 model for YLD (0.18–0.36) and PROT (0.13–0.46). The performance of the MTME model for these two traits likely relates to the lower genetic trait correlations and lower correlation among the environments for these traits in 2018–19 (Supplementary Figure 4). Analogous to YLD and PROT, the MTME model resulted in a higher prediction accuracy than the ST-CV1 model for TW, HT, and HDs in 2018–19 (Figure 3). For instance, the average PA using MTME for TW, HT, and HDs was 0.42, 0.42, and 0.36, respectively, which translates to an improvement of 19, 68, and 12%, respectively. Furthermore, the PA using the MTME model outstripped the ST-CV1 model in all the five environments for TW (0.32–0.52) and HT (0.41–0.54), and in four environments for HDs (Figure 3).

In contrast to 2018–19, we observed higher genetic correlations among the five traits and higher environmental correlations in 2019–20 (Supplementary Tables 2, 3 and Supplementary Figure 4). As a result of high correlation values, we observed a consistent improvement in the PA of MTME in all the environments for all the five traits (Figure 4 and Supplementary Table 4). For YLD, the MTME model also performed better than the single-trait model in most of the environments, except HYS. The average PA for YLD using the MTME model was 0.43, which was 22% better than the ST-CV1 model. Furthermore, the MTME model appeared to be superior for predicting PROT and TW (Figure 4). For PROT, the MTME model performed best in all the locations, with a PA ranging from 0.52 to 0.67 (Supplementary Table 5). We achieved an improvement in PA of up to 100% (OND) using the MTME model (0.52) over the single-trait model (0.26) with 71% improvement on average. The PA for TW was higher using the MTME model than the other models, ranging from 0.53 to 0.67, with a mean improvement of 17% over the ST-CV1 model (Supplementary Table 5). Similarly, the average PA of the MTME model was the highest for HT (0.49) and HDs (0.53), which outstrips the ST-CV1 model by 48 and 51%, respectively.

### Application of MTME Model in the Breeding Program

**Figure 5:**
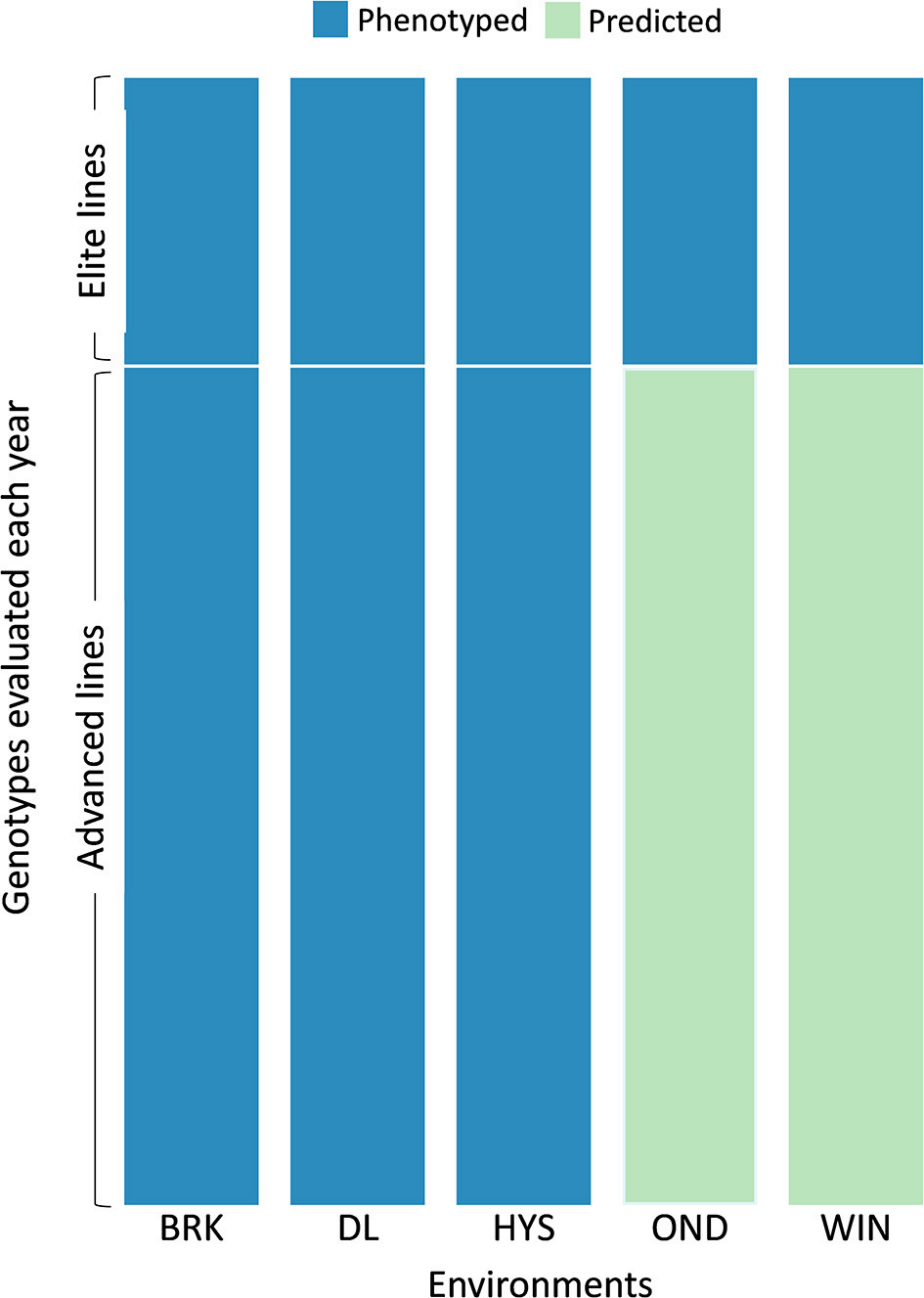
Testing design for the independent prediction of agronomic traits using the MTME model. Each year, a set of elite and advanced lines is evaluated over multiple locations. The sparse testing design proposes phenotyping of elite lines in all the environments (five in this scenario) and advanced lines in fewer environments (three in this scenario). For independent prediction, the dataset from 2018–19 comprised 55 elite lines with checks and 96 advanced lines. The 2019–20 dataset comprised 42 elite lines with checks and 114 advanced lines. Five environments: BRK, Brookings; DL, Dakota Lakes; HYS, Hayes; OND, Onida; and WIN, Winner.

Based on the cross-validation results, we evaluated the efficacy of the MTME model in reducing phenotypic efforts in the breeding program. We used the MTME model to estimate the GEBV of advanced lines in environments where only elite lines are evaluated. In the tested allocation design, we used phenotypic data of EYTs from five environments and AYTs from three environments to predict GEBVs of AYTs in remaining environments (Figure 5). Two environments, OND and WIN, were used as testing environments for predicting AYTs. For 2018–19, we predicted the performance of 96 AYT lines, whereas 2019–20 comprised a prediction of 114 AYT lines in two environments. Table 2 elucidates the predictive ability for the five agronomic traits using MTME in an independent prediction scenario. Moderate PA was observed for all the traits in both environments except for WIN in 2019–20. For OND, the results showed a better prediction accuracy than WIN for YLD and TW. Overall, the results suggest that the MTME model could be used by evaluating an overlapping set of lines over multiple environments and lines in early testing could be tested in fewer environments.

**Table 2:** Predictive ability for the independent prediction of advanced breeding lines (AYTs) in new environments using the MTME model.

**Year**	**Env^a^**	**Predictive ability^b^**				
		**Grain yield**	**Grain protein**	**Test weight**	**Plant height**	**Days to heading**
2018–19	OND	0.44	0.37	0.43	0.49	0.27
	WIN	0.30	0.25	0.38	0.30	0.46
2019–20	OND	0.36	0.27	0.44	0.22	0.41
	WIN	0.15	0.32	0.25	0.18	0.24

*Tables shows Pearson correlation between the observed and predictive values of agronomic traits in the AYTs in two different environments over two growing seasons*.

a *Env refers to different trial location. BRK, Brookings; DL, Dakota Lakes; HYS, Hayes; OND, Onida; and WIN, Winner*.

b *The predictive ability for five agronomic traits using the MTME model in independent prediction of advanced lines. Refer to Figure 5 for the design of the prediction scheme*.

## Discussion

In recent years, genomic prediction has been intensively evaluated in wheat breeding programs to select and advance lines for several traits of interest (Rutkoski et al., 2014, 2016; Haile et al., 2020; Juliana et al., 2020). However, improving the PA of complex traits remains a challenge for successfully implementing GS in breeding programs. The choice and optimization of statistical models are crucial to improve the performance of GS. Most plant breeding programs currently rely on univariate genomic prediction models to target a single trait at a time. An advantage of multivariate prediction approaches over single-trait models that have been demonstrated in some recent studies is utilizing correlations between multiple traits and environments (Jia and Jannink, 2012; Sun et al., 2017; Lado et al., 2018; Ward et al., 2019; Ibba et al., 2020). This study evaluated the application of multi-trait and multi-environment prediction models to predict five key traits of varying genetic architecture across diverse environments in a breeding program.

The ridge-regression best linear unbiased prediction (rrBLUP) is one of the most often used single-trait prediction models. The rrBLUP has an advantage over Bayesian models in predicting complex traits governed by several loci with small effects (Lorenz et al., 2011). We used rrBLUP as a baseline model (ST-CV1) for comparison with different multivariate approaches. The PA for agronomic traits using ST-CV1 was comparable with other studies using the same model (Pérez-Rodríguez et al., 2012; Charmet et al., 2014; He et al., 2016; Maulana et al., 2021). For instance, the PA for YLD was between 0.13 and 0.43 for 2018–19 and 0.27 and 0.5 for 2019–20. The PA for TW in both growing seasons was higher than the PA for other traits because of the highly heritable nature of this trait (Figures 3, 4).

We evaluated the multi-trait model using two cross-validation schemes. The first scheme (MT-CV1) conducts multi-trait prediction for new un-phenotyped individuals, and the testing set has not been phenotyped for any of the traits. In the second cross-validation scheme (MT-CV2), phenotype information for the predicted trait is missing, whereas phenotype information for the secondary traits is available in the testing set (Lado et al., 2018; Bhatta et al., 2020). In this study, the PA of the MT-CV1 model was found similar to that of the ST-CV1 model for most of the trait-environment combinations in both growing seasons (Supplementary Tables 4, 5). Several studies have reported marginal or no improvement with MT-CV1, where information from secondary traits is limited to the training set (Calus and Veerkamp, 2011; Lado et al., 2018; Schulthess et al., 2018; Arojju et al., 2020; Bhatta et al., 2020). However, other studies reported an improvement in GP when the MT-CV1 model included secondary traits with moderate-high heritability (Jia and Jannink, 2012; Rutkoski et al., 2012; Guo et al., 2014). Jia and Jannink, 2012 suggested that the MT-CV1 approach might be more useful when the primary trait has very low heritability (*H*^2^<*0.2*). In this study, the similarity in performance of the MT-CV1 and ST-CV1 models might be contributed by the moderate to high heritability estimated for most of the traits and the small size of the training population.

In contrast to MT-CV1, the MT-CV2 model significantly improved the PA for all agronomic traits in all the environments, suggesting that the inclusion of secondary traits in the training and testing sets improves the predictive performance of complex traits (Supplementary Tables 4, 5). Several studies have reported a similar improvement in prediction using the MT-CV2 model for agronomic and end-use quality traits in wheat (Rutkoski et al., 2016; Sun et al., 2017; Lado et al., 2018), rice (Wang et al., 2017), barley (Bhatta et al., 2020), sorghum (Fernandes et al., 2018), and ryegrass (Arojju et al., 2020). The MT-CV2 model outperformed the single-trait model for YLD prediction in all environments. However, the extent of improvement using the MT-CV2 model varied with traits and environments tested. As multi-trait models rely on the genetic correlation between traits (Calus and Veerkamp, 2011; Jia and Jannink, 2012), differences in prediction improvements due to the MT-CV2 model can be attributed to the varying degrees of genetic correlations observed in different environments. We observed a high genetic correlation among the traits in 2019–20 that resulted in a higher prediction accuracy for the different traits in this growing season (Figure 1 and Supplementary Tables 2, 3). The results suggest that MT-CV2 could likely be very useful if we can include data for HT, HDs, and other spectral indices recorded using a high throughput method for predicting YLD. In addition, the MT-CV2 approach could be really useful to predict hard-to-phenotype end-use quality traits by the inclusion of already available agronomic data for the testing set.

We also evaluated the BMTME model (referred to as MTME) that generalizes a multi-trait model to consider the correlations among multiple environments. Recently, two studies reported an increase in the PA of agronomic and end-use quality traits in wheat using the BMTME approach (Guo et al., 2020; Ibba et al., 2020). Because of the different training process, we did not directly compare the MTME model with the MT-CV2 model but compared both with the ST-CV1 model. In 2018–19, the MTME model proved to be better than the ST-CV1 and MT-CV1 models for all the traits except YLD and PROT. However, the MTME model outperformed the ST-CV1 and MT-CV1 models in 2019–20 for all the traits in all the environments (Supplementary Table 5). The mean improvement in PA (across five environments) using MTME model over the ST-CV1 reached up to 19, 71, 17, 48, and 51% for YLD, PROT, TW, HT, and HDs, respectively. The differences in performance of the MTME model in 2019–20 compared with 2018–19 relate to the observed genetic correlations among the traits as well as among the environments in these growing seasons (Supplementary Figure 2A). As discussed earlier, the genetic correlations between traits and correlation among environments were higher in 2019–20 compared with those in 2018-19 Thus, a higher PA was observed for the traits showing a high correlation among the different environments. For example, the five environments were highly correlated for PROT (0.56–0.76) compared with YLD (0.23–0.65) (Supplementary Figures 3, 4), explaining the difference in the improvement of PA for these traits. Overall, the results suggest that the MTME model could be successfully applied in a program if there is a moderate to high correlation for a trait between environments and overcome the effect of a small training population.

Apart from the statistical model, the heritability (*H*^2^) of a trait is another crucial factor for improving PA (Lorenz et al., 2011; Combs and Bernardo, 2013). Several studies have found that low heritability often results in lower prediction accuracy in single-trait genomic prediction (Heffner et al., 2009; Jannink et al., 2010). The application of multi-trait models can improve the PA of low-heritability traits using the information from correlated traits with high heritability (Jia and Jannink, 2012; Jiang et al., 2015; Lado et al., 2018; Bhatta et al., 2020). The heritability estimates for most of the traits in different environments were moderate to high in this study, with few exceptions. The use of the MT-CV2 model significantly improved the predictive ability for PROT in WIN (0.15 to 0.29) and TW in DL (0.23 to 0.39), where highly heritable and moderately correlated traits were included in the model. In contrast, the MT-CV2 model did not improve the PA for HDs in HYS (0.23 to 0.25), as the primary trait was weakly correlated to the highly heritable secondary traits in the model. The results suggest that the inclusion of highly heritable but weakly correlated secondary traits in the multi-trait model may not improve the PA.

Genomic prediction has been suggested to implement sparse testing in multi-environment trials and reduce the resources involved in phenotyping (Jarquin et al., 2020). Based on the promising cross-validation results using MTME models, we evaluated the application of this model in the breeding program to reduce phenotyping resources. At the SDSU winter wheat breeding program, we evaluate a set of elite (EYTs) and advanced (AYTs) lines each year in multiple environments. However, the results suggest that GP models developed using phenotypic data from all locations of EYTs and limited locations of AYTs can predict AYTs in remaining environments (Table 2). This strategy could be useful as we evaluate ~40 EYTs and ~110 AYTs each year in replicated nurseries and testing the AYT plots at two/three locations instead of five can save substantial resources. Though we used this strategy to predict AYTs at two locations, further improved GP models assisted with environics data can help to predict more environments with better accuracy. Moreover, this strategy can be expanded to predict preliminary breeding lines at earlier testing stages.

In conclusion, this study evaluated the PA of univariate and multivariate GP models for five agronomic traits in advanced winter wheat breeding lines. We compared two different cross-validation strategies mocking practical breeding scenarios. Overall, the results supported the practical implementation of multivariate GS models in predicting complex traits. We found a significant advantage of using MT and MTME models when correlated traits and/or environments are included in the models. The results suggest that the inclusion of correlated traits and environments in prediction models can offset the limitation of a small training population, allowing the use of advanced breeding lines to predict preliminary breeding lines in the same year or the following one. It will be interesting to further study the inclusion of different combinations of secondary traits in the MT model to increase the PA of YLD. We envision that the evaluation of secondary traits such as plant height, tillers/m^2^, spike length, and spike density that have high correlations with YLD using an unmanned aerial system (UAS) in winter wheat yield trials could help predict YLD. This would permit trials on a large number of locations (e.g., >10) but harvesting only in a limited number (e.g., 2–3) of locations. Similarly, evaluating secondary traits (grain protein, flour protein, water absorption, gluten content, and quality) could facilitate the prediction of other complex traits such as end-use quality. Finally, GS holds a tremendous potential for improving the selection accuracy of complex traits in wheat breeding; however, we believe GEBVs will complement phenotyping efforts rather than replacing them. Future breeding strategies should focus on increasing the efficiency of breeding programs by maximizing genetic gain.

## Data Availability Statement

The datasets presented in this study can be found in online repositories. The names of the repository/repositories can be found in the article/Supplementary Material.

## Author Contributions

SS and HG conceptualized the experiment and designed the methodology. SS, HG, CH, NB, JH, and JZ performed the field trials. HG and SS performed the data curation, performed the data analysis, software implementation, and visualization, and wrote the original manuscript. PA, AB, and GB performed genotyping and SNP discovery. TR assisted in data analysis and software implementation. GB, PA, EO, BT, JH, and SA contributed to the interpretation of results and revision of the manuscript. SS obtained the funding for the project. All authors approved the manuscript.

## Conflict of Interest

The authors declare that the research was conducted in the absence of any commercial or financial relationships that could be construed as a potential conflict of interest.

## Publisher's Note

All claims expressed in this article are solely those of the authors and do not necessarily represent those of their affiliated organizations, or those of the publisher, the editors and the reviewers. Any product that may be evaluated in this article, or claim that may be made by its manufacturer, is not guaranteed or endorsed by the publisher.
