# Supplementary material for: Multi-Trait Multi-Environment Genomic Prediction of Agronomic Traits in Advanced Breeding Lines of Winter Wheat
Source: Front Plant Sci. 2021 Aug 18;12:709545. doi: 10.3389/fpls.2021.709545 (PMC8416538; doi:10.3389/fpls.2021.709545)

SUPPLEMENTARY MATERIAL

Multi-trait multi-environment genomic prediction of agronomic traits in advanced breeding lines of winter wheat

Harsimardeep S Gill[1], Jyotirmoy Halder[1], Jinfeng Zhang[1], Navreet K Brar[1], Teerath S Rai[1], Cody Hall[1], Amy Bernardo[2], Paul St Amand[3], Guihua Bai[3], Eric Olson[4], Shaukat Ali[1], Brent Turnipseed[1], Sunish K Sehgal[1]*

[1]Department of Agronomy, Horticulture & Plant Science, South Dakota State University, Brookings, SD, USA
[2]Department of Plant Pathology, Kansas State University, Manhattan, KS, USA.
[3]USDA-ARS, Hard Winter Wheat Genetic Research Unit, Manhattan, KS, USA
[4]Department of Plant, Soil and Microbial Sciences, Michigan State University, East Lansing, MI, USA

***Corresponding Author:**
Sunish K. Sehgal,
Department of Agronomy, Horticulture & Plant Science, South Dakota State University, Brookings, SD 57007
Email address: sunish.sehgal@sdstate.edu

The genomic datasets used in this study can be found online repositories at https://github.com/SunishSehgal/.

Supplementary Table S1. Information of the experimental sites used in the growing seasons of 2018-19 and 2019-20.

**Site**	**Coordinates**	**2018-19**		**2019-20**	
		**Date seeded**	**Date harvested**	**Date seeded**	**Date harvested**
Brookings (BRK)	44°18'35.3"N 96°40'14.5"W	9/16/2018	8/6/2019	9/20/2019	7/20/2020
Dakota Lakes (DL)	44°17'34.2"N 99°59'40.6"W	9/28/2018	7/23/2019	9/19/2019	7/17/2020
Hayes (HYS)	44°22'24.8"N 101°02'45.1"W	9/14/2018	7/31/2019	9/17/2019	7/21/2020
Onida (OND)	44°42'57.5"N 100°23'04.2"W	9/25/2018	8/1/2019	9/18/2019	7/28/2020
Winner (WIN)	43°29'57.0"N 99°51'58.4"W	10/2/2018	7/25/2019	9/27/2019	7/15/2020

Supplementary Table S2. Genetic correlation between five agronomic traits evaluated in 2018-19 estimated using the BMTME model. Evaluated traits include grain yield (YLD); grain protein content (PROT); test weight (TW); plant height (HT); and days to heading (HD).

**Trait**	**YLD**	**PROT**	**TW**	**HT**	**HD**
YLD	1	-0.15	0.29	0.00	-0.13
PROT	-0.15	1	0.35	-0.01	0.09
TW	0.29	0.35	1	-0.02	0.07
HT	0.00	-0.01	-0.02	1	-0.01
HD	-0.13	0.09	0.07	-0.01	1

Supplementary Table S3. Genetic correlation between five agronomic traits evaluated in 2019-20 estimated using the BMTME model. Evaluated traits include grain yield (YLD); grain protein content (PROT); test weight (TW); plant height (HT); and days to heading (HD).

**Trait**	**YLD**	**PROT**	**TW**	**HT**	**HD**
YLD	1	-0.44	-0.14	-0.43	-0.18
PROT	-0.44	1	0.25	0.38	-0.14
TW	-0.14	0.25	1	0.39	-0.09
HT	-0.43	0.38	0.39	1	0.18
HD	-0.18	-0.14	-0.09	0.18	1

Supplementary Table S4. Prediction accuracy for five traits recorded at five different environments in 2018-19 using different genomic prediction models (ST-CV1, single-trait model; MT-CV1, multi-trait model with CV1 scheme; MT-CV2, multi-trait model with CV2 scheme; MTME, multi-trait multi-environment model with CV1 scheme). The value in bold indicates the best performing model for given trait at respective location.

**Trait**	**Env**	**ST-CV1**		**MT-CV1**		**MT-CV2**		**MTME**	
		**Mean PA**	**S.E.**	**Mean PA**	**S.E.**	**Mean PA**	**S.E.**	**Mean PA**	**S.E.**
Yield	Brookings	0.28	0.005	0.29	0.02	**0.56**	0.02	0.26	0.03
	Dakota Lakes	0.32	0.004	0.28	0.03	**0.40**	0.02	0.36	0.02
	Hayes	0.38	0.004	0.35	0.02	**0.41**	0.02	0.25	0.03
	Onida	**0.43**	0.004	0.42	0.01	**0.43**	0.02	0.35	0.03
	Winner	0.13	0.005	0.03	0.02	0.15	0.03	**0.18**	0.03
	Average	0.31	-	0.27	-	0.**39**	-	0.28	-
Protein content	Brookings	0.40	0.004	0.41	0.02	**0.45**	0.02	0.33	0.03
	Dakota Lakes	0.50	0.004	**0.56**	0.02	0.51	0.02	0.45	0.03
	Hayes	0.32	0.004	0.34	0.02	**0.38**	0.02	0.26	0.03
	Onida	0.39	0.004	0.41	0.01	0.39	0.02	**0.46**	0.03
	Winner	0.15	0.004	0.20	0.02	**0.29**	0.02	0.13	0.04
	Average	0.35	-	0.38	-	0.40	-	0.32	-
Test weight	Brookings	0.31	0.004	0.31	0.02	**0.48**	0.02	0.35	0.03
	Dakota Lakes	0.23	0.005	0.23	0.01	**0.39**	0.02	0.32	0.02
	Hayes	0.50	0.004	0.49	0.02	**0.54**	0.02	0.52	0.02
	Onida	0.43	0.005	0.41	0.02	0.40	0.02	**0.47**	0.03
	Winner	0.35	0.005	0.36	0.02	0.32	0.02	**0.43**	0.02
	Average	0.36	-	0.36	-	0.42	-	0.42	-
Plant height	Brookings	0.26	0.005	0.24	0.02	0.38	0.02	**0.44**	0.03
	Dakota Lakes	0.16	0.005	0.16	0.02	0.38	0.02	**0.41**	0.04
	Hayes	0.33	0.004	0.21	0.02	0.31	0.02	**0.42**	0.03
	Onida	0.16	0.004	0.16	0.02	0.27	0.02	**0.45**	0.03
	Winner	0.32	0.004	0.30	0.02	0.34	0.02	**0.54**	0.03
	Average	0.25	-	0.21	-	0.34	-	0.42	-
Heading date	Brookings	0.46	0.004	0.46	0.03	0.44	0.03	**0.49**	0.02
	Dakota Lakes	0.33	0.005	0.32	0.02	**0.47**	0.02	0.40	0.04
	Hayes	0.23	0.005	0.24	0.02	**0.25**	0.02	0.16	0.04
	Onida	0.35	0.005	0.39	0.02	**0.40**	0.01	0.37	0.03
	Winner	0.35	0.005	0.35	0.02	**0.37**	0.02	0.37	0.03
	Average	0.34	-	0.35	-	0.38	-	0.36	-

Supplementary Table S5. Prediction accuracy for five traits recorded at five different environments in 2019-20 using different genomic prediction models (ST-CV1, single-trait model; MT-CV1, multi-trait model with CV1 scheme; MT-CV2, multi-trait model with CV2 scheme; MTME, multi-trait multi-environment model with CV1 scheme). The value in bold indicates the best performing model for given trait at respective location.

**Trait**	**Env**	**ST-CV1**		**MT-CV1**		**MT-CV2**		**MTME**	
		**Mean PA**	**S.E.**	**Mean PA**	**S.E.**	**Mean PA**	**S.E.**	**Mean PA**	**S.E.**
Yield	Brookings	0.29	0.006	0.27	0.03	**0.52**	0.02	0.39	0.03
	Dakota Lakes	0.33	0.005	0.33	0.02	**0.57**	0.02	0.46	0.03
	Hayes	0.50	0.004	0.52	0.02	**0.71**	0.01	0.44	0.02
	Onida	0.44	0.003	0.41	0.02	**0.50**	0.02	0.46	0.03
	Winner	0.27	0.005	0.23	0.02	**0.67**	0.01	0.43	0.02
	Average	0.36	-	0.35	-	0.**59**	-	0.43	-
Protein content	Brookings	0.41	0.005	0.41	0.02	0.58	0.02	**0.62**	0.02
	Dakota Lakes	0.34	0.004	0.36	0.02	0.59	0.01	**0.67**	0.02
	Hayes	0.40	0.004	0.40	0.02	0.56	0.02	**0.59**	0.02
	Onida	0.26	0.005	0.22	0.02	0.31	0.02	**0.52**	0.03
	Winner	0.34	0.004	0.35	0.02	**0.66**	0.01	0.58	0.02
	Average	0.35	-	0.35	-	0.54	-	0.60	-
Test weight	Brookings	0.56	0.003	0.57	0.01	0.59	0.01	**0.64**	0.02
	Dakota Lakes	0.58	0.004	0.58	0.02	0.61	0.01	**0.63**	0.02
	Hayes	0.58	0.004	0.57	0.02	0.64	0.01	**0.67**	0.02
	Onida	0.60	0.003	0.60	0.02	0.59	0.02	**0.66**	0.02
	Winner	0.37	0.005	0.33	0.02	0.50	0.02	**0.53**	0.02
	Average	0.54	-	0.53	-	0.59	-	0.63	-
Plant height	Brookings	0.35	0.004	0.35	0.02	0.40	0.02	**0.51**	0.02
	Dakota Lakes	0.31	0.005	0.26	0.02	0.43	0.02	**0.44**	0.02
	Hayes	0.43	0.004	0.41	0.02	0.53	0.02	**0.59**	0.02
	Onida	0.28	0.005	0.24	0.03	0.43	0.02	**0.48**	0.02
	Winner	0.28	0.005	0.27	0.02	0.36	0.02	**0.41**	0.03
	Average	0.33	-	0.31	-	0.43	-	0.49	-
Heading date	Brookings	0.37	0.004	0.41	0.02	0.42	0.02	**0.58**	0.02
	Dakota Lakes	0.29	0.004	0.27	0.02	0.30	0.02	**0.54**	0.02
	Hayes	0.29	0.005	0.36	0.02	0.32	0.03	**0.48**	0.04
	Onida	0.44	0.004	0.46	0.02	0.45	0.02	**0.56**	0.02
	Winner	0.35	0.004	0.31	0.02	0.39	0.02	**0.48**	0.02
	Average	0.35	-	0.36	-	0.38	-	0.53	-

Supplementary Figure S1. Illustration of different cross-validation schemes used to evaluate different genomic prediction models.

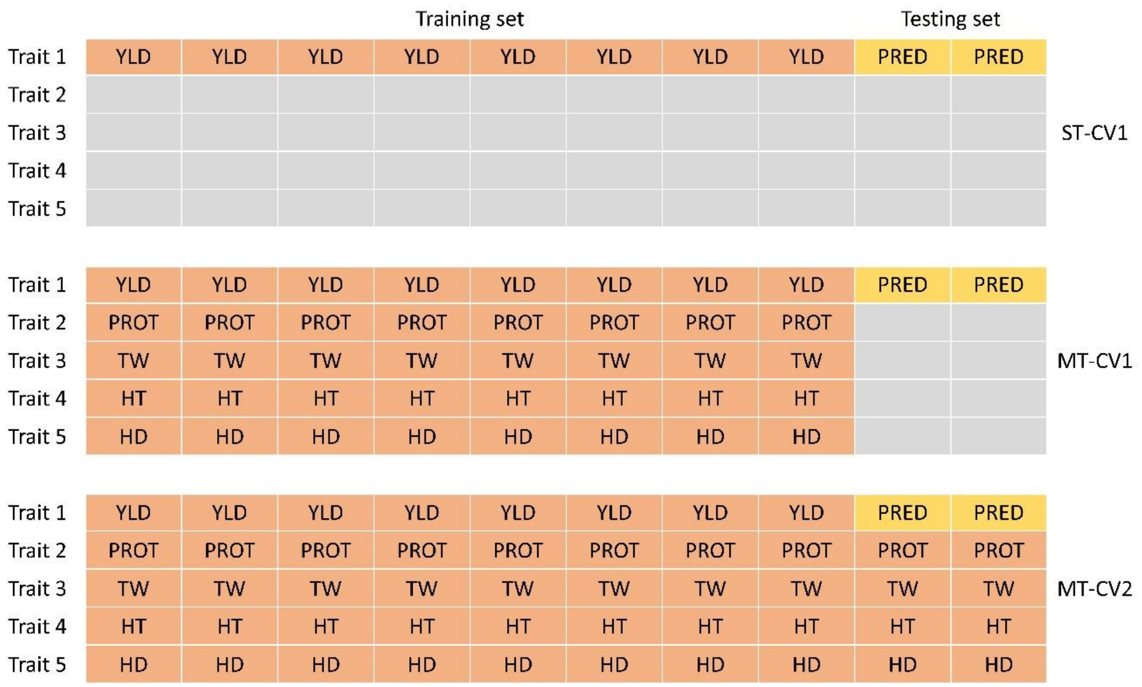

Supplementary Figure S2. Correlation coefficients among five agronomic traits recorded at five locations in 2018-19. Evaluated traits include YLD, grain yield; PROT, grain protein content; TW, test weight; HT, plant height; and HD, days to heading.

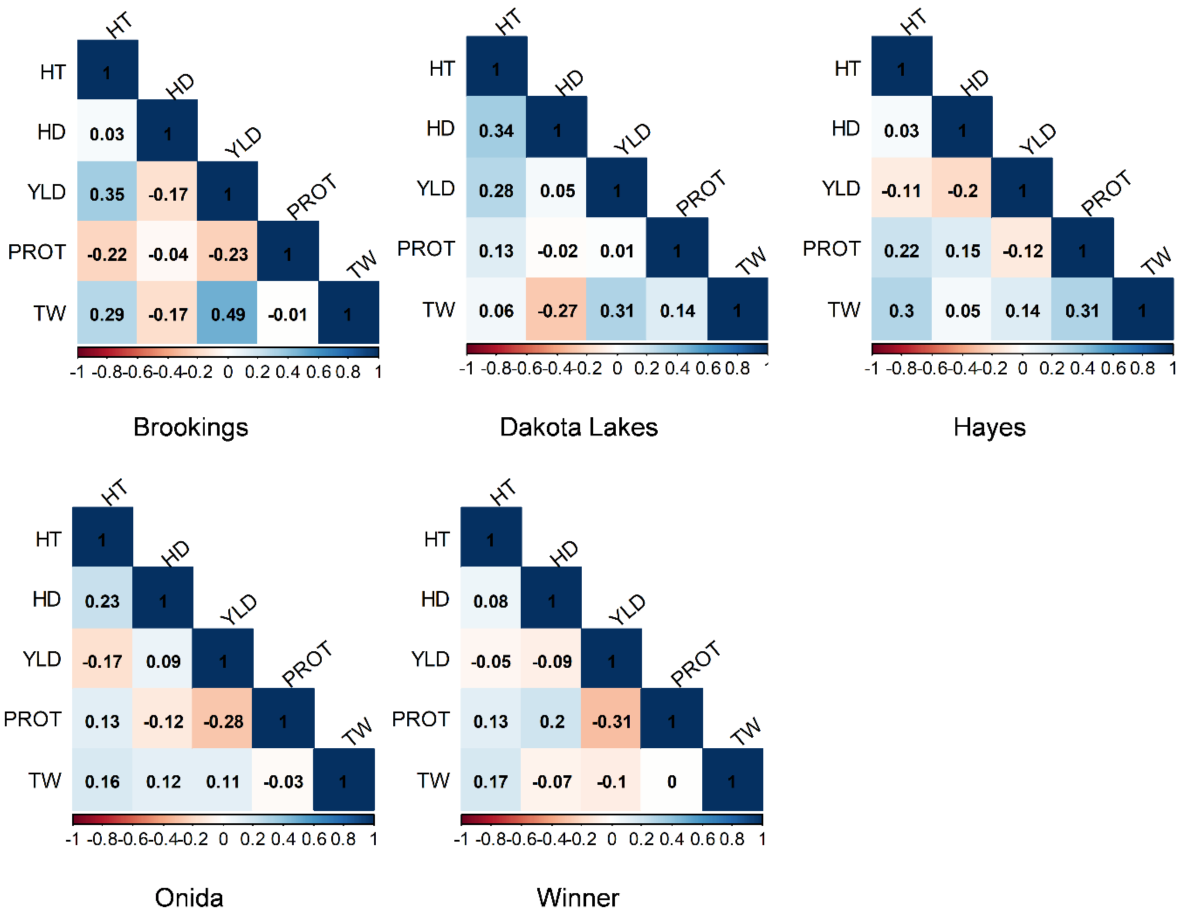

Supplementary Figure S3. Correlation coefficients for five agronomic traits evaluated at five locations in 2019-20. Evaluated traits include grain yield (YLD); grain protein content (PROT); test weight (TW); plant height (HT); and days to heading (HD).

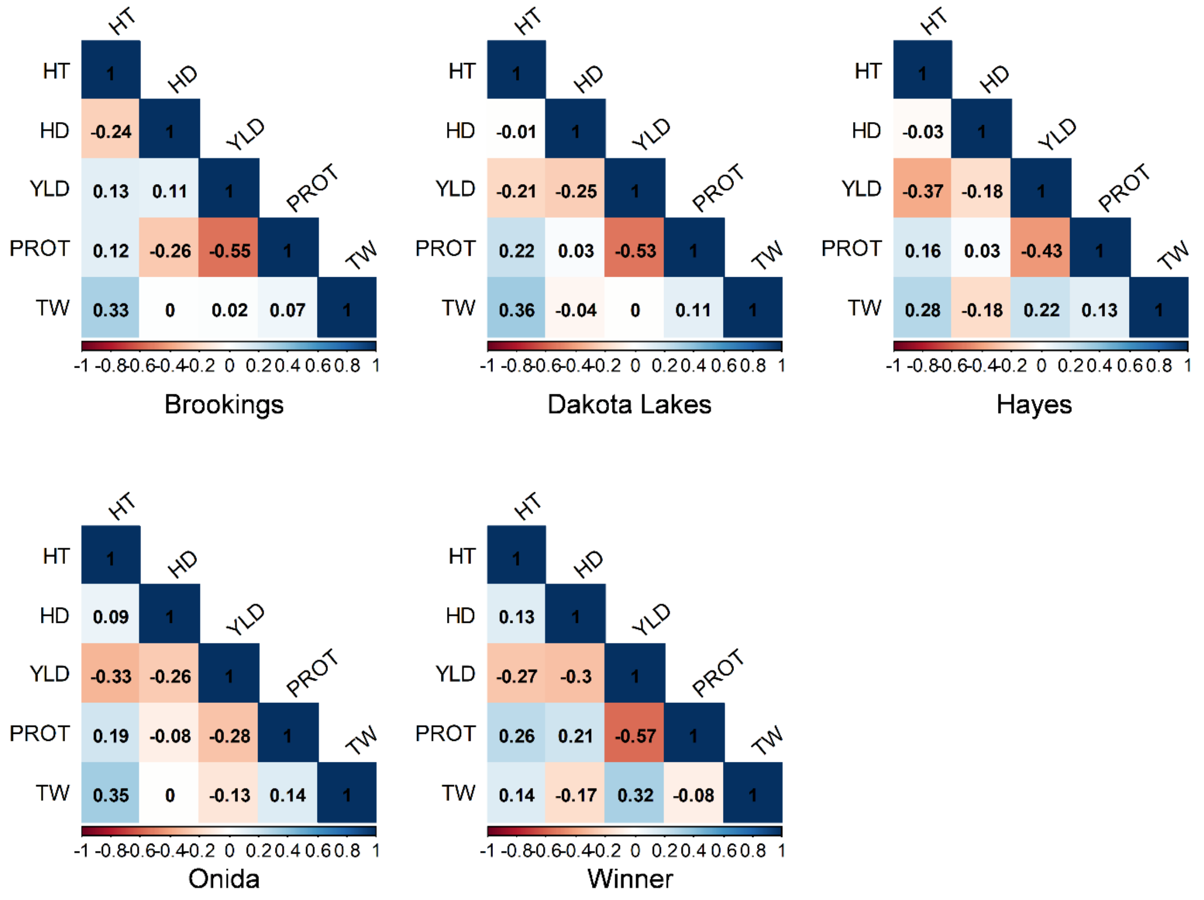

Supplementary Figure S4. Correlation coefficients among five environments (Brookings, BRK; Dakota Lakes, DL; Hayes, HYS; Onida, OND; and Winner, WIN) for five traits evaluated in (**A**) 2018-19 and (**B**) 2019-20.

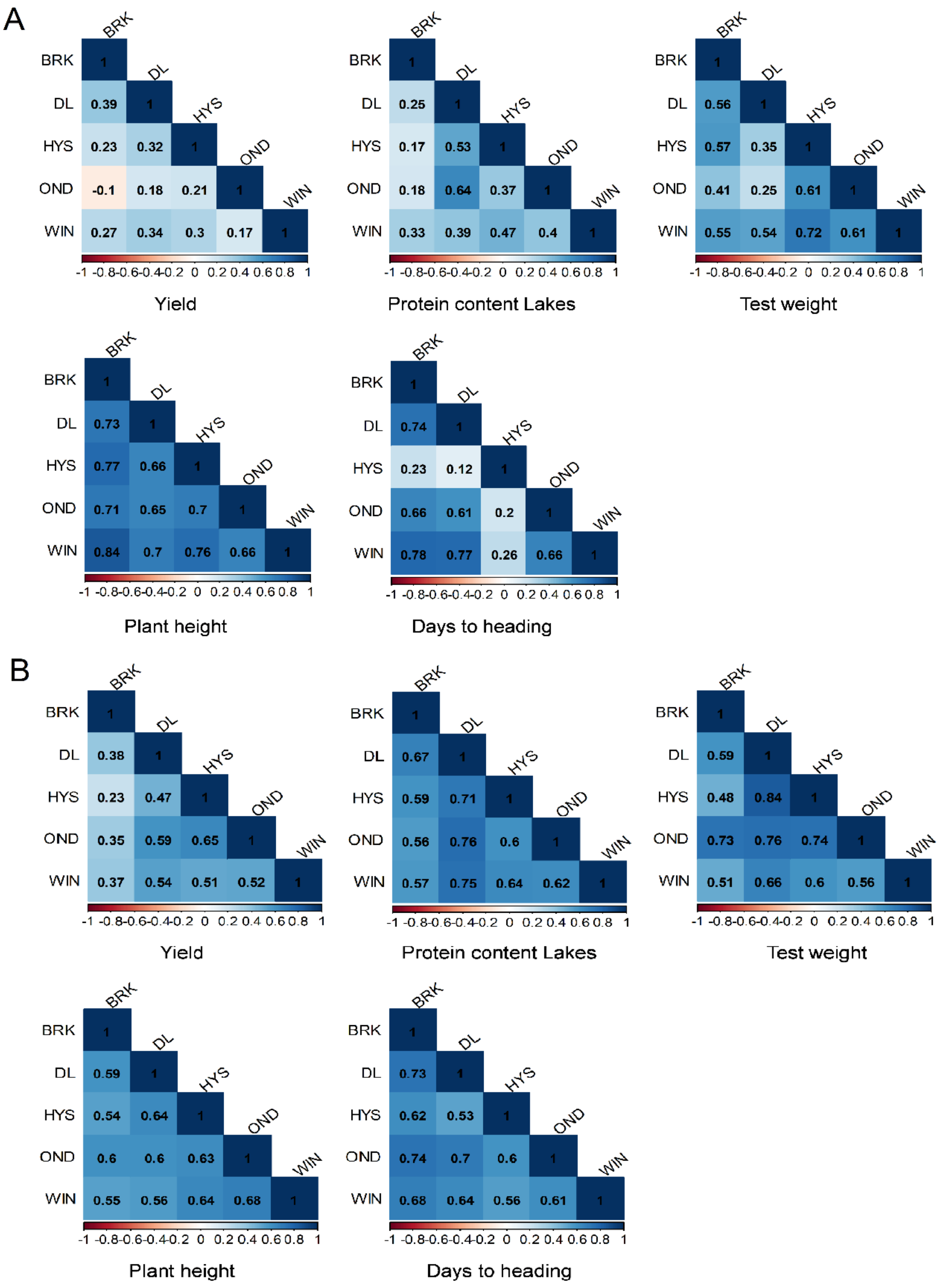

Supplementary Figure S5. Heatmap of the kinship matrix using 10,294 SNPs **(A)** for 151 lines evaluated in the growing season of 2018-19, and **(B)** for 156 lines evaluated in the growing season of 2019-20.

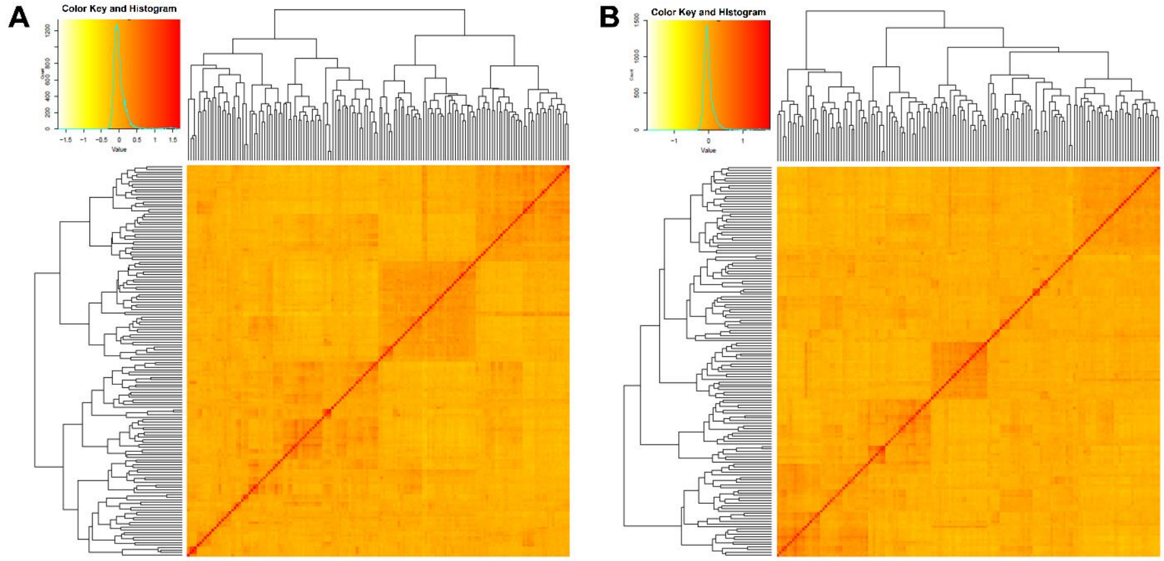

Supplement: Supplementary file 1 [file Data_Sheet_1.pdf]
